# Cost-Effectiveness of a Community Pharmacist-Led Sleep Apnea Screening Program – A Markov Model

**DOI:** 10.1371/journal.pone.0063894

**Published:** 2013-06-21

**Authors:** Clémence Perraudin, Marc Le Vaillant, Nathalie Pelletier-Fleury

**Affiliations:** 1 Faculté de Médecine Paris-Sud Paris XI, Le Kremlin-Bicêtre, France; 2 Centre de Recherche Médecine, Sciences, Santé, Santé Mentale, Société (CERMES3), UMR 8211– INSERM U988, Villejuif, France; Central Queensland University, Australia

## Abstract

**Background:**

Despite the high prevalence and major public health ramifications, obstructive sleep apnea syndrome (OSAS) remains underdiagnosed. In many developed countries, because community pharmacists (CP) are easily accessible, they have been developing additional clinical services that integrate the services of and collaborate with other healthcare providers (general practitioners (GPs), nurses, etc.). Alternative strategies for primary care screening programs for OSAS involving the CP are discussed.

**Objective:**

To estimate the quality of life, costs, and cost-effectiveness of three screening strategies among patients who are at risk of having moderate to severe OSAS in primary care.

**Design:**

Markov decision model.

**Data Sources:**

Published data.

**Target Population:**

Hypothetical cohort of 50-year-old male patients with symptoms highly evocative of OSAS.

**Time Horizon:**

The 5 years after initial evaluation for OSAS.

**Perspective:**

Societal.

**Interventions:**

Screening strategy with CP (CP-GP collaboration), screening strategy without CP (GP alone) and no screening.

**Outcomes measures:**

Quality of life, survival and costs for each screening strategy.

**Results of base-case analysis:**

Under almost all modeled conditions, the involvement of CPs in OSAS screening was cost effective. The maximal incremental cost for *“screening strategy with CP”* was about 455€ per QALY gained.

**Results of sensitivity analysis:**

Our results were robust but primarily sensitive to the treatment costs by continuous positive airway pressure, and the costs of untreated OSAS. The probabilistic sensitivity analysis showed that the “*screening strategy with* CP” was dominant in 80% of cases. It was more effective and less costly in 47% of cases, and within the cost-effective range (maximum incremental cost effectiveness ratio at €6186.67/QALY) in 33% of cases.

**Conclusions:**

CP involvement in OSAS screening is a cost-effective strategy. This proposal is consistent with the trend in Europe and the United States to extend the practices and responsibilities of the pharmacist in primary care.

## Introduction

Obstructive sleep apnea syndrome (OSAS) is a common disease. According to epidemiological studies, the prevalence of OSAS is 5% to 10% in the general population [1]–[3]. The disorder is associated with high morbidity and mortality rates because of cardiovascular diseases and traffic accidents [4]. When OSAS is left untreated, there are direct medical costs associated with the disease, and comorbidities [5], and indirect costs that are associated with vigilance disorders (i.e., traffic accidents, work accidents, losses in productivity, and absenteeism) [6], [7].

OSAS screening and management remain an issue. A 2006 Ministry of Health report estimated that only 15% of French patients with OSAS are diagnosed [8]. In the United States, 30% to 40% of adult patients who visit a primary care clinician have a high risk of having sleep apnea, but most patients do not discuss their sleep-related symptoms, and less than a third of patients at high risk of having OSAS have sleep-related symptoms documented in their medical records [9]. This phenomenon is likely to increase because of difficulties with GP recruitment and retention in deprived areas [10], [11].

In the United States, Canada, and several European countries, the role of the community pharmacist (CP) as the healthcare professional that traditionally dispenses doctors' prescriptions has changed. Because CP are easily accessible, they have been developing additional clinical services that integrate the services of and collaborate with other healthcare providers (i.e., general practitioners (GP), nurses, etc.) [12]–[14]. In France, the “*Hospital, Patients, Health and Territories*” (HPST) healthcare reform law was adopted in 2009. This law broadens the role of the CP in providing these services. The CP has new responsibilities in front-line healthcare, healthcare coordination, screening and therapeutic education (article 38 of the HPST law). This context leads to the development of alternative strategies for primary care screening programs that involve the CP.

The purpose of this study was to assess the cost-effectiveness of a CP-led sleep apnea screening program (in collaboration with GPs) among patients who are at risk of having moderate to severe OSAS. The “*screening strategy with CP*” was compared with “*screening strategy without CP”* and “*no screening”*.

## Methods

### Decision Model Structure

**Figure 1:**
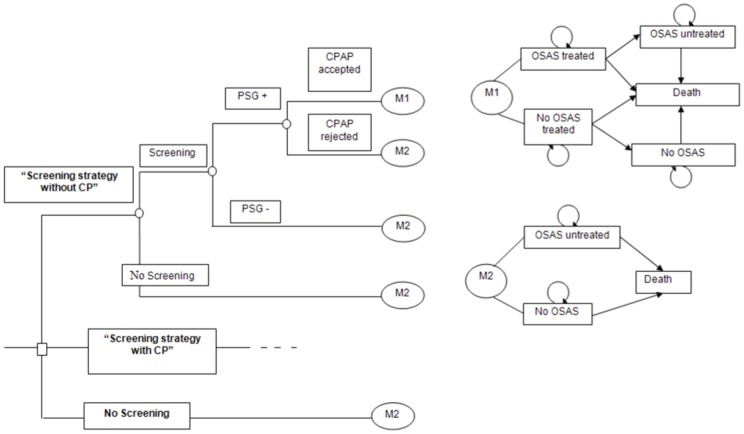
Analytic Decision Tree and Markov model of patients according to screening and treatment status.

We constructed a decision-analytic Markov model. The decision tree is described in Figure 1. Three strategies were compared.

“*No screening*”
“*Screening strategy without CP*”: we assumed that 15% of patients who were at risk for OSAS who visited a GP were referred to a sleep specialist [8]. GPs usually refer patients to a sleep specialist on an ad hoc basis due to symptoms.
“*Screening strategy with CP*”: patients were screened for OSAS by the CP before visiting their GP. They were informed of an OSAS screening program through flyers that were placed on the counter. The CP identified 50-year-old male or older with 3 symptoms highly evocative of OSAS (snoring, obesity and hypertension) and convinced them to consult their GP for further investigation. Indeed, the pre-test probability of having OSAS is usually high in this population (see below). After recruitment, the CP program involved a discussion with the patient on the risks and comorbidities associated with untreated OSAS and 2 validated questionnaires for OSAS screening, including the Berlin Questionnaire and the Epworth Sleepiness Scale [15], [16]. The Berlin questionnaire, given the way it is scored, had 100% chance to have 2 or more positive categories (high likelihood of having OSAS) in this population. The Epworth score might vary, but it was in no case, if low, an indication not to pursue investigations. In this scenario, together with a letter to the GP, explaining that the patients had symptoms highly evocative of OSAS, patients were asked to communicate their scores to the GP to encourage a referral to a sleep specialist. The CP could also call the doctor. We assumed that some of the patients who received a CP program did not keep their GP informed. This source of uncertainty was taken into account in the different screening rates that were further tested in the model.

The screening strategies with and without the CP implied that the GP referred a number of patients who were at risk for OSAS to a sleep specialist who prescribed a diagnostic test. According to the diagnostic results, a continuous positive airway pressure (CPAP) treatment was proposed. When patients exited the screening and diagnostic evaluation, they were entered into a Markov model. The Markov model included 4 mutually exclusive and collectively exhaustive health states: (1) OSAS treated, (2) OSAS untreated, (3) No OSAS, and (4) No OSAS treated (false-positive diagnosis and accepted CPAP). Based on the probability of transition at each cycle, individuals could stay in the same health state or move to a new health state. The patients who were in the OSAS treated or no OSAS treated states moved to the OSAS untreated or no OSAS health states if they discontinued CPAP use. The analytical time horizon was 5 years (the Markov cycle length was 1 year), which is consistent with the period in which data on the long-term effectiveness of and compliance with CPAP are currently available.

The cost-effectiveness analysis was assessed through the standard metric of the incremental cost-effectiveness ratio (ICER). Health outcomes were expressed in Quality-Adjusted Life Years (QALYs), and costs were considered from a societal perspective and are expressed in Euros. Consistent with standard practices in health economic analyses, we discounted costs and QALYs at an annual rate of 3% to incorporate a time preference for money and good health. We used TreeAge Pro 2012 (TreeAge Software Inc., Williamstown, MA) for the analysis.

### Data inputs

#### Sources of data

Data were derived from published literature. The model parameters are summarized in Table 1.

**Table 1:** Key Model Assumptions.

Variables	Base case	Range	References	Distribution
**Cohort characteristics**				
Sex	Male	-	-	No distribution assigned
Age (years)	50	-	-	No distribution assigned
**OSAS pretest probability**	0.82	[0.79–0.85]	[17], [18]	No distribution assigned
**Diagnostic test**				
PSG specificity	1.00	[0.90–1.00]	[17]	No distribution assigned
PSG sensitivity	0.97	[0.90–1.00]	[19]	No distribution assigned
**Mortality risk** Odds Ratio for mortality risk from cardiovascular disease in patients with untreated OSAS	2.87	IC95 [1.17–7.51]	[21]	No distribution assigned
**Treatment CPAP**				
Initial rate of refusal	0.102	[0–0.204]	[22]	Beta distribution α = 1.15;β = 10.15
Annual rate of cessation				
Year 1	0.10	[0–0.20]	[22]	No distribution assigned
Years 2–5	0.06	[0–0.12]	[22]	No distribution assigned
**Utilities**				
No OSAS	0.435	σ = 0.17*	[25], [26]	Beta distribution α = 3.7;β = 4.8
No OSAS treated	0.32	σ = 0.17*	[25], [26]	Beta distribution α = 2.4;β = 5.1
OSAS untreated	0.32	σ = 0.17*	[25], [26]	Beta distribution α = 2.4;β = 5.1
OSAS treated	0.55	σ = 0.26	[25], [26]	Beta distribution α = 2.01;β = 1.65
**CP rates of screening**	0.454	[0.25–0.65]	unpublished data	Uniform distribution
**Discount rate**	3%	[0%–5%]	-	No distribution assigned
**Cost estimates in Euros**				
**Diagnosis**				
In-hospital PSG	€837	[±20%]	Health insurance fund	Triangular distribution
GP visits	€23	-	Health insurance fund	No distribution assigned
Specialist visits	€50	-	Health insurance fund	No distribution assigned
**CPAP treatment**				
CPAP per year	€1200	[€420–€1400]	Health insurance fund, [19], [27]	Triangular distribution
**Annual Specialist Visit**				
Year 1 (per year)	€75	-		-
Years 2–5 (per year)	€25	-	Health insurance fund	-
**CP intervention per patient**	€75	[€25–€125]	Estimation	Triangular distribution
**Costs of untreated OSAS**	€1800	[€441–€2714]	[28], [30]	Triangular distribution

* We assumed that the 0.17– standard deviation of the utility for untreated OSAS was equivalent for no OSAS and no OSAS treated.

(€1 =  $US 1.30).

#### Population characteristics

The base case population was a cohort of 50-year-old male patients with symptoms highly evocative of OSAS, such as frequent snoring, excessive daytime sleepiness and co-morbidities (obesity and hypertension). The patients could be healthy or affected by OSAS. In this cohort, the pre-test probability of having moderate to severe OSAS (AHI≥15) was estimated to be 82% [17], [18].

#### Screening rates associated with the CP program

Actually, no study, except one has yet assessed the effectiveness of a community pharmacist-led sleep apnea screening program. Results indicate a screening rate at 45.4% [*unpublished data*]. The protocol was approved by a local ethical committee (*Comité de Protection des Personnes Ile-de-France V n° 11838*). Written informed consent was obtained from all participants. In the sensitivity analysis we tested screening rates from 25% to 65%.

#### Diagnostic tests

We chose polysomnography (PSG) at a hospital as the referent diagnostic test. The sensitivity and specificity of the PSG test were collected from the literature [17], [19].

#### Baseline rate of events and increased risk associated with OSAS

The age- and gender-specific all-cause mortality was estimated using “2007–2009 France life tables”, which were released by the National Institute of Demographic Studies (INED) [20]. The death rates of patients with untreated OSAS were adjusted according to the higher risk [17] i.e., the risk associated with cardiovascular events: odds ratio of 2.87 (1.17–7.51) [21], which was derived from a cohort of individuals with severe OSAS (AHI>30). This could lead to an overestimation of death rates of patients in our fictive cohort.

#### CPAP compliance

In a literature review, Engleman et al [22] indicated that the rate of initial refusal of CPAP therapy varies from 5 to 50% with a mean (standard deviation) of 10.2% (0.14). As a baseline, we chose 10%. The cumulative curve for CPAP discontinuation over time approximated an exponential decay function. The curve had an initially steep fall-off that levels after 3–4 years. According to the same study, we assumed an annual rate of treatment discontinuation of 10% in the first year, and 6% per year thereafter [22]. In the sensitivity analysis, we tested models of perfect compliance, which assumed that all of the patients accepted CPAP and continued treatment for 5 years, and low compliance, which doubled the rate of patients who never used therapy and doubled the rate of CPAP cessation.

#### Utility parameters

QALYs are the sum of the durations of health states multiplied by the mean utility of each of the health states [23]. In patients with OSAS, 2 studies have determined utilities with the standard gamble technique [24], [25]. We used the utilities that were determined by Chakravorty et al., as these values were derived from a larger sample size in a prospective manner. Patients with untreated and treated OSAS had utility values of 0.32 and 0.55, respectively. CPAP treatment was associated with an improvement in utility of 0.23. As suggested by Chervin et al, patients without OSAS were assigned a utility value of 0.43 that was midway between the utilities for treated and untreated OSAS, and patients without OSAS but treated wrongly were assigned a similar utility value than those with untreated OSAS, i.e., 0,32 [26].

### Costs and resource use

Costs are presented in Table 1. Costs are expressed in Euros (Currently €1 =  $US 1.30).

The costs of the diagnostic process included the initial GP consultation, two sleep specialist consultations (before and after the PSG), and a PSG test at a hospital. The base case costs were derived from health insurance fund databases (€837). In the sensitivity analysis, we tested the cost of PSG in the range of 20% to account for variation in hospital costs.

The annual cost of CPAP treatment included the price that was set by the health insurance fund, which included a CPAP rental, accessories and maintenance, and the costs of follow-up visits. The follow-up visits included 3 specialist visits for the first year (at 1 month, 6 months and 1 year) and annual follow-up visits. In the sensitivity analysis, we used current prices that have been established in other countries. In Spain, Mar et al (2003) estimated the minimum cost of CPAP treatment to be €420/year, and this cost can increase to €1400/year in the United States (Costs were converted using exchange rates and prices current in 2012) [18], [27].

The costs of CP program were divided into fixed costs, which included the purchase of equipment (privacy area and software) and pharmacist training, and variable costs, which included costs associated with the pharmacist's time in providing screening program and the purchase of materials (i.e., leaflets and questionnaires). A training program for pharmacists could consist of one talk-workshop (focused on the illness, the identification of patients at risks and treatment options) and of provision of up-to-date information on OSAS. For our analysis, the cost of CP program was defined by expert opinion. We estimated a total cost of €75 per patient at baseline, and we used upper and lower limits of €25 and €125 per patient in the sensitivity analysis.

Untreated OSAS patients have a higher healthcare utilization than treated patients [28], [29]. For untreated OSAS, a Danish study estimated the additional direct medical costs to be €441 per patient per year [30]. The costs included in-hospital costs, outpatient costs, drug treatment and primary healthcare and private practice specialist costs. Few studies have quantified the indirect costs that are related to untreated OSAS, but the costs are substantial [31]. Jennum (2009) estimated maximum indirect costs €2273 per patient per year [30]. We assumed a mean direct and indirect cost of €1800 for untreated OSAS. In the sensitivity analysis, we tested a minimum cost of €441 (only additional direct medical costs) and a maximum cost of €2714 per patient per year (direct and indirect costs).

### Sensitivity Analysis

We performed two types of sensitivity analysis. First, we used a deterministic 1-way sensitivity analysis by systematically varying 1 parameter at a time across wide intervals. Then, we used a probabilistic sensitivity analysis by simultaneously varying several model parameters using a second-order Monte Carlo technique. The probabilistic sensitivity analysis allowed us to understand the aggregate impact of uncertainties in the model's underlying parameters on the overall cost-effectiveness estimates; the method involves randomly sampling model parameter values that are drawn from joint probability distributions to estimate the expected costs and effects of alternatives scenarios. The analysis is repeated over large numbers of samples (10,000 iterations) to produce a distribution of cost-effectiveness ratios. This method allows the estimation of the probability that a strategy is dominant (i.e., more effective and less costly) or that it is more effective but more costly. We adopted the commonly cited value of society's willingness to pay €40,000 for a QALY. We specified the distributions for the model variables to represent uncertainty in the estimates (Table 1). Beta distributions were provided for all of the conditional probabilities and utilities. For costs, triangular distributions were used with upper and lower limits that we determined. A uniform distribution was chosen for the CP screening rate with the underlying assumption that a patient who had received the CP program had a random probability (between 25% and 55%) of being referred to a sleep specialist, depending on the GP's motivation.

## Results

### Base Case

The cost-effectiveness ratios are presented in Table 2. The “*No screening*” strategy was less costly than the other strategies but was also less effective.

**Table 2:** Cost-effectiveness ratio.

	No screening	Screening strategy without CP	Screening strategy with CP				
			Screening rate: 25%	Screening rate: 35%	Screening rate: 45%	Screening rate: 55%	Screening rate:65%
**Expected costs**	€6652.28	€6603.76	€6631.07	€6583.38	€6535.70	€6488.01	€6440.33
**QALYs**	1.55	1.64	1.70	1.77	1.83	1.89	1.95
**CE ratios**	€4291.79/Q	€4026.69/Q	€3900.63/Q	€3719.42/Q	€3571.42/Q	€3432.81/Q	€3302.73/Q

Cost-Effectiveness ratios (CE ratio) are expressed in Euros per QALY (€/Q).

**Figure 2:**
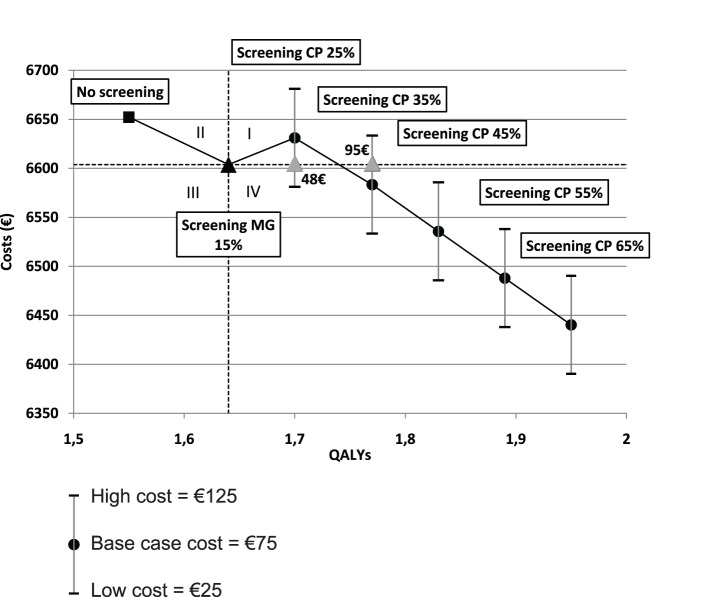
Costs of different strategies according to their effectiveness and the CP intervention cost. The strategies included in quadrant I were more effective and more costly than “*Screening strategy without CP*”. The strategies included in quadrant II were less effective and more costly (dominated); the strategies included in quadrant III were less effective and less costly; and the strategies included in quadrant IV were more effective and less costly (dominant). The slope of the line between any two points represents the incremental cost-effectiveness ratio for two pathways.

Figure 2 shows the costs of the different screening strategies according to their effectiveness and different CP screening rates. For a CP screening rate of 25%, the incremental cost-effectiveness ratio of the “*Screening strategy with CP*” compared with the “*Screening strategy without CP*” was €455.17 per QALY gained. The “*Screening strategy with CP*” dominated the “*Screening strategy without CP*” only if the CP program cost was less than €48. For a CP screening rate of 35%, the “*Screening strategy with CP*” dominated the “*Screening strategy without CP*” in the base case. The “*Screening strategy with CP*” became more costly only if the CP program cost was more than €95. For a CP screening rate of 45% or higher, the “*Screening strategy with CP*” was in quadrant IV, i.e., it dominated the “*Screening strategy without CP*” for any CP program cost.

### Univariate Sensitivity Analysis

The one-way sensitivity analyses suggest that the results from the base case are robust (Table 3). In all of the analyses and considering different screening rates, the “*Screening strategy with CP*” dominated the “*Screening strategy without CP*”, either because it was more effective and less costly or because it was within the range of cost-effectiveness (ICER = €6186.67/QALY). The results were primarily sensitive to the CPAP costs and the costs of untreated OSAS.

**Table 3:** The results of the one-way sensitivity analysis.

Scenarios	Screening strategy without CP	Screening strategy with CP				
		Screening rate: 25%	Screening rate: 35%	Screening rate: 45%	Screening rate: 55%	Screening rate: 65%
**OSAS probability [Base case = 0.82]**						
Low: 0.79	-	ICER = €553.17/Q	dominant	dominant	dominant	dominant
High: 0.85	-	ICER = €357.33/Q	dominant	dominant	dominant	dominant
**PSG Specificity [Base case = 1.00]**						
Low: 0.90	dominated	dominant	dominant	dominant	dominant	dominant
**PSG Sensitivity [Base case = 0.97]**						
Low: 0.90	-	ICER = €625.50/Q	dominant	dominant	dominant	dominant
High: 1.00	-	ICER = €382.33/Q	dominant	dominant	dominant	dominant
**Compliance with CPAP treatment**						
Low	-	ICER = €1653.25/Q	ICER = €715.75/Q	ICER = €372.23/Q	ICER = €232.41/Q	ICER = €145.90/Q
High	dominated	dominant	dominant	dominant	dominant	dominant
**Discount rate [Base case = 3%]**						
No: 0%	-	ICER = €351.67/Q	dominant	dominant	dominant	dominant
High: 5%	-	ICER = €31.03/Q	dominant	dominant	dominant	dominant
**Cost of in-hospital PSG [Base case = €837]**						
Low: −20%	-	ICER = €176.83/Q	dominant	dominant	dominant	dominant
High:+20%	-	ICER = €735.17/Q	ICER = €101.69/Q	dominant	dominant	dominant
**Cost of CPAP treatment [Base case = €1200]**						
Low:€420	dominated	dominant	dominant	dominant	dominant	dominant
High:€1400	-	ICER = €1318.00/Q	ICER = €639.69/Q	ICER = €459.16/Q	ICER = €365.28/Q	ICER = €307.74/Q
**Cost of CP intervention [Base case = €75]**						
Low:€25	dominated	dominant	dominant	dominant	dominant	dominant
High: €125	-	ICER = €1288.50/Q	ICER = €227.85/Q	dominant	dominant	dominant
**Cost of untreated OSAS [Base case = €1800]**						
Low: €441	-	ICER = €6186.67/Q	ICER = €5133.85/Q	ICER = €5071.58/Q	ICER = €5039.20/Q	ICER = €5019.35/Q
High:€2714	dominated	dominant	dominant	dominant	dominant	dominant

Incremental Cost-Effectiveness ratios (ICER) are expressed in Euros per QALY gained (€/Q).

(€1 =  $US 1.30).

### Probabilistic Sensitivity Analysis

**Figure 3:**
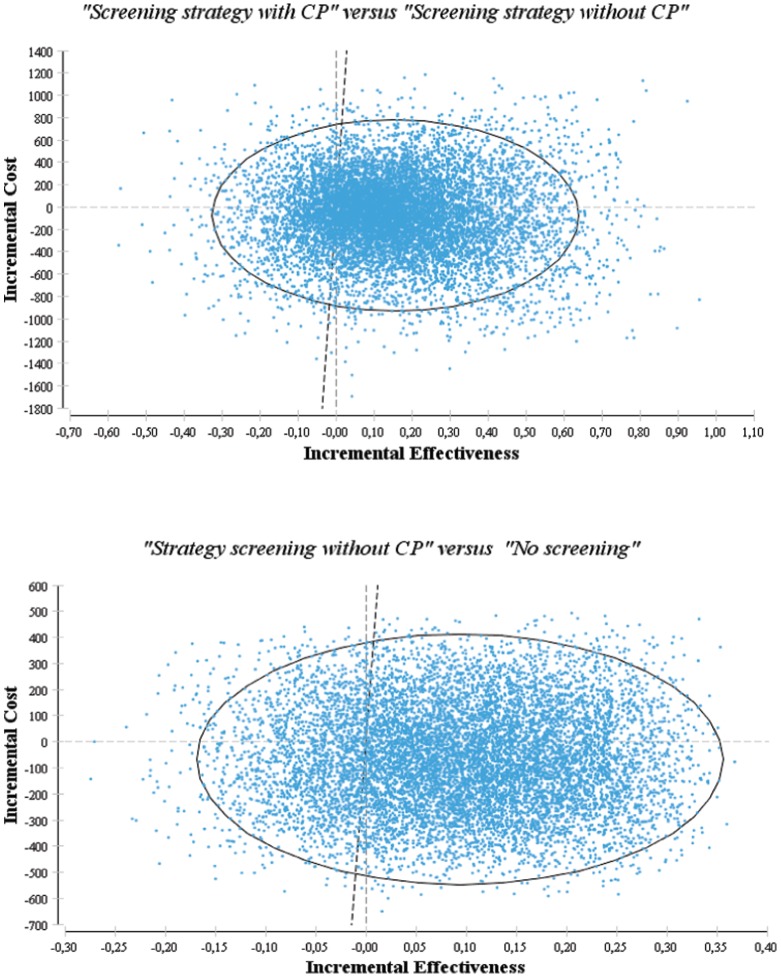
Incremental cost-effectiveness for the 10 000 Monte Carlo simulations on the cost-effective plane. The scatterplots show the distribution of the incremental cost-effectiveness ratio for the different strategies. The ellipses show the central 95th percentile of each distribution. The overlaid lines have slopes that are equal to the willingness-to-pay threshold (€40000).

The results are presented in Figure 3. OSAS screening in primary care was attractive, as the “*Screening strategy without CP*” was more effective and less costly than the “*No screening*” strategy (i.e., dominant) for 51% of the simulations and was more effective and more costly (but below the acceptability threshold) for 29% of the simulations. The “*Screening strategy with CP*” was more effective and less costly than the “*Screening strategy without CP*” for 47% of the simulations, and it was more effective and more costly but below the acceptability threshold for 33% of the simulations.

### Comment

To our knowledge, this study is the first to evaluate a CP program to improve OSAS screening, in collaboration with GPs, in at-risk individuals. This study shows that CP involvement represents an efficient use of healthcare resources. Compared with the screening strategy involving only the GP, the “*Screening strategy with CP*” was dominant in 80% of cases. In other words, the “*Screening strategy with CP*” was more effective and less costly, or it was within the cost-effective range (maximum ICER = €6186.67/QALY).

These results are conditional on many assumptions, especially about model parameters values. The only way to validate these assumptions and costs savings results would be to collect actual data from an experimental study which integrate outcomes both health and cost savings. Here, sensitivity analyses allow to test the robustness of results for a wide range of situations.

CP involvement in OSAS screening in primary care is an alternative strategy that is consistent with current trends in pharmaceutical care and the extension of CP responsibilities in primary care. Faced with an increasing demand for healthcare and reductions in services that are offered – particularly by GPs – questions are being asked in various regions of France about the healthcare principles of proximity, availability and access. Healthcare reform was adopted in 2009 (known as the “*Hospital, Patients, Health and Territories*” law) to broaden the role of pharmacists in providing these services. After primarily being a dispenser of medications, the pharmacist is now being assigned responsibilities in front-line healthcare, healthcare coordination, screening and therapeutic education (article 38 of the HPST law). The United States and the United Kingdom also face issues concerning the extension of the scope of practice in pharmacy, pharmaceutical care and access to primary care. The Medicare Modernization Act of 2003 (effective in 2006) recognized the pharmacist as a provider of Medication Therapy Management (MTM) services for the elderly [32]. In the United Kingdom, Medicines Use Review (MUR) involves a face-to-face consultation with the patient to improve patient knowledge and prescribed medicine use, with a particular focus on enhancing adherence [33]. In this favorable context for experimentation in primary care organization, it appeared to be appropriate to evaluate an OSAS screening program in community pharmacies.

Intervention studies involving CPs are not new, particularly in the field of screening and monitoring cardiovascular diseases. These studies have demonstrated the feasibility and effectiveness of CP services [34], [35]. Only three studies in Switzerland [36] and Australia [37], [38] have analyzed the development of a screening program for sleep disorders in community pharmacies. The authors were enthusiastic about the feasibility of these experiments and concluded that the pharmacist, through the establishment of a screening program on a targeted population, could improve the identification of patients who are at risk of OSAS. However, none of these studies performed an economic analysis. In our study, the base case for CP screening rate was based on a French pilot study performed in experimental setting. Because this screening rate could be different in routine conditions, our extensive sensitivity analysis allowed us to highlight the conditions in which the intervention would be cost-effective.

This study has several limitations. First, our analysis considered indirect costs for untreated OSAS, and our results, although robust, were sensitive to variation in these costs. We should be cautious about our findings, as only one study [30] has documented indirect costs. For this reason, we used a wide range of costs in the sensitivity analysis.

Second, we used utilities that were determined from the standard gamble technique in patients with OSAS [25]. This approach may conflict with the societal perspective and may have created a bias for the estimates. Several studies have used utilities derived from the EuroQol5D (EQ-5D), but the EQ-5D is a generic instrument that is not sensitive to changes in health states in patients with OSAS [39]. Therefore, there can be little appreciable improvement with CPAP treatment [27].

Third, our analysis was limited to a hypothetical population of 50-year-old male patients with hypertension who were at risk of having moderate to severe OSAS (AHI≥15). Transition probabilities and data were based on published studies that included patients with similar characteristics. Therefore, our model may not be applicable to older or younger patients or to patients with different pre-test risks. However, this cohort of patients is the most likely to benefit from screening OSAS.

In conclusion, we showed that CP involvement in a screening protocol of patients who are at risk of OSAS is a cost-effective strategy. This proposal is consistent with the trend in Europe and the United States to extend the practices and responsibilities of the pharmacist in primary care.
